# Dental Caries diagnosis from bitewing images using convolutional neural networks

**DOI:** 10.1186/s12903-024-03973-9

**Published:** 2024-02-10

**Authors:** Parsa ForouzeshFar, Ali Asghar Safaei, Foad Ghaderi, Sedighe Sadat Hashemikamangar

**Affiliations:** 1 Department of Data Science, Faculty of Mathematical Sciences, Tarbiat Modares University, Tehran, Iran; 2 Department of Medical Informatics, Faculty of Medical Sciences, Tarbiat Modares University, Tehran, Iran; 3 Department of Data Science, Faculty of Interdisciplinary Science and Technology, Tarbiat Modares University, Tehran, Iran; 4 Human-Computer Interaction Lab, Electrical and Computer Engineering Department, Tarbiat Modares University, Tehran, Iran; 5 Restorative Department, Dental school, Tehran University of Medical Sciences, Tehran, Iran

**Keywords:** Dental caries, Bitewing images, Convolutional neural Network (CNN), Diagnosis, Classification, Tooth decay, Dental health

## Abstract

**Background:**

*Dental caries*, also known as *tooth decay*, is a widespread and long-standing condition that affects people of all ages. This ailment is caused by bacteria that attach themselves to teeth and break down sugars, creating acid that gradually wears away at the tooth structure. Tooth discoloration, pain, and sensitivity to hot or cold foods and drinks are common symptoms of tooth decay. Although this condition is prevalent among all age groups, it is especially prevalent in children with baby teeth. Early diagnosis of dental caries is critical to preventing further decay and avoiding costly tooth repairs. Currently, dentists employ a time-consuming and repetitive process of manually marking tooth lesions after conducting radiographic exams. However, with the rapid development of artificial intelligence in medical imaging research, there is a chance to improve the accuracy and efficiency of dental diagnosis.

**Methods:**

This study introduces a data-driven model for accurately diagnosing dental decay through the use of Bitewing radiology images using convolutional neural networks. The dataset utilized in this research includes 713 patient images obtained from the Samin Maxillofacial Radiology Center located in Tehran, Iran. The images were captured between June 2020 and January 2022 and underwent processing via four distinct Convolutional Neural Networks. The images were resized to 100 × 100 and then divided into two groups: 70% (4219) for training and 30% (1813) for testing. The four networks employed in this study were AlexNet, ResNet50, VGG16, and VGG19.

**Results:**

Among different well-known CNN architectures compared in this study, the VGG19 model was found to be the most accurate, with a 93.93% accuracy.

**Conclusion:**

This promising result indicates the potential for developing an automatic AI-based dental caries diagnostic model from Bitewing images. It has the potential to serve patients or dentists as a mobile app or cloud-based diagnosis service (clinical decision support system).

## Introduction

According to the World health organization (WHO), dental caries (tooth decay) is defined as the destruction of the enamel layer of the tooth by acids produced by the action of bacteria on sugar [1]. The impact of poor dental and oral fitness on children’s quality of life cannot be overstated [2]. Early detection of caries in its incipient stages is paramount, as it can prevent patients from undergoing further invasive treatment procedures such as extensive restorations and root canal therapy (RCT). Given the significant implications of advanced caries for children, it is essential to prioritize early detection and treatment to minimize the need for costly and potentially painful interventions later on. The diagnosis of caries lesions has conventionally involved visual and tactile detection, alongside bitewing radiography [3]. The interpretation of the radiographic appearance of caries lesions in bitewing radiography may lead to improved diagnostic accuracy. Interpretation of bitewing radiography can be time-consuming for dentists in their daily dental practice, and different examiners often have different judgments. This bias may cloud the judgments of various dentists. Moreover, a simple examination can be a burden for patients too. Panoramic, periapical, and bitewing X-rays are three common types of radiographs that are widely used in clinical practice.

Nowadays Machine Learning is widely used for Object Recognition, Pattern Recognition, Natural Language Processing, and image processing tasks [4]. In the field of medical image processing, several works have been done. Deep learning, a subfield of machine learning, has shown great potential in various image recognition and classification tasks, including medical image analysis [5]. However, it has gained significant popularity in recent years due to its ability to achieve amazing results, even at human-level performance [6]. In [7], a deep learning model was developed for the classification of COVID-19 based on CT images. Convolutional neural network (CNN) is one of the most popular architectures of Deep Learning networks [8]. The main advantage of CNN compared to its predecessors is that it automatically detects significant features without any human supervision which makes it the most used [9]. Recent advancements in artificial intelligence have made it possible to diagnose dental caries via machine learning techniques, with a particular focus on neural networks and deep learning. This development is highly significant; as traditional diagnosis methods can often result in dentists misidentifying healthy teeth as carious (false positives) or decayed teeth as healthy (false negatives). Additionally, the availability of dentists to diagnose caries rapidly may be limited, underscoring the importance of leveraging AI in this domain. In this article, we will delve into how artificial neural networks and deep learning can be leveraged for the accurate diagnosis of tooth caries from radiographic images of teeth. Lian et al., [10], used to detect caries lesions, classify different radiographic extensions on panoramic films, and compare the classification results with those of expert dentists. Experts evaluated 1160 dental panoramic films to detect and classify caries lesions based on depth. The study used no new net (nnU-Net) for segmentation and DenseNet121 for classification. Results showed high accuracy and recall rates for both techniques, and one of the positive points of this study was that they followed both segmentation and classification techniques. A study by Faria et al. [11] has introduced a method that uses artificial intelligence neural network to detect and predict regular caries or radiation-related caries (RRC) in head and neck cancer patients undergoing radiotherapy. The study analyzed 420 teeth retrospectively from 15 HNC patients using PyRadiomics, and an artificial neural network classifier (ANN) was utilized to analyze the data. The proposed method demonstrated a sensitivity of 98.8% in detecting RRC and predicted an RRC with 99.2% accuracy. This study presented a new perspective on dental caries, however, its smaller sample size compared to other studies may limit its impact. Lee et al. [12] used 3000 periapical radiographic images to train a pre-trained GoogLeNet Inception v3 CNN network for processing and transfer learning. The diagnostic accuracies for premolar, molar, and both were found to be 89.0, 88.0, and 82.0%, respectively. The premolar model achieved an AUC of 0.917, the molar model achieved an AUC of 0.890, and both premolar and molar models achieved an AUC of 0.845 using the deep CNN algorithm. This study utilized a large dataset and applied its models to various teeth. Sornam et al., [13], a different approach was used. They used Linearly Adaptive Particle Swarm Optimization [LA-PSO] and Back Propagation Neural Network for the classification of dental caries based on the features that have been extracted from the Panoramic X-ray images. They achieved a 99% accuracy. Their method was novel, however it is hard to this model in other studies. In this study, the combination of statistics and neural networks can be seen which is an important feature. A combination of CNN and LSTM networks known as CNN-LSTM was suggested by Singh et al. [14] . The aim was to classify dental caries according to the G.V. black classes. The optimal CNN-LSTM model proposed achieved a 96% accuracy rate. Mao et al. [3], used CNNs to classify restorations and caries. They implemented transfer learning CNNs on Bitewing films by dint of Gaussian high-pass filter and Otsu’s threshold image enhancement technology. In the study, four networks were evaluated for their effectiveness in restoration and caries diagnosis. AlexNet achieved an accuracy of 95.56%, making it a valuable tool for computer-aided diagnosis in dentistry. Moran et al. [15], utilized Inception and ResNet networks with three different learning rates (0.1, 0.01, 0.001), and after 2000 iterations, the Inception model with a 0.001 learning rate achieved the best results. The accuracy on the test set was 73.3%. This study detected both caries and restorations which was worth noticing. Their accuracy was not too high which can be challenging. Mertens et al. [16], found that an AI-powered diagnostic-support software for detecting proximal caries in bitewing radiographs helped dentists increase their sensitivity and mean area under the Receiver-Operating- Characteristics curve. Their method was new and it compared the AI methods with dentists diagnosis power. In a study by Bayraktar et al. [17], they assessed the effectiveness of using CNNs to diagnose interproximal caries lesions in digital bitewing radiographs. They analyzed 1000 bitewing images and found 11,521 approximal surfaces through augmentations. The YOLO algorithm was used for detection. The CNN model showed an overall accuracy of 94.59%, with a sensitivity of 72.26%, specificity of 98.19%, PPV of 86.58%, and NPV of 95.64%. Using YOLO was an important feature of this study, however, there was a gap between sensitivity value and other criteria reported in their study. Bayrakdar et al., [18], an AI system called CranioCatch was used to detect and segment dental caries on 621 bitewing radiographs using VGG16 and U-net models. The results showed high rates of sensitivity, precision, and F-measure for caries detection and segmentation. The AI models outperformed 5 experienced observers on an external dataset which is an important achievement. A study was conducted by Canas et al. [19] to evaluate the reliability of a web-based AI program for detecting interproximal caries in bitewing radiographs. They analyzed 300 images using a convolutional neural network and calculated various metrics such as accuracy rate, sensitivity, specificity, positive and negative predictive value, positive and negative likelihood ratio, and areas under the receiver operating characteristic curves. Imak et al. [20] have proposed a new method for detecting dental caries using a multi-input deep convolutional neural network ensemble model. The approach involves pre-processing, deep convolutional neural network, and score-based fusion phases. The team used pre-learned weights of the AlexNet architecture and a transfer learning approach to adapt this architecture in dental caries detection. The study analyzed 340 periapical images from 310 patients and achieved an impressive accuracy rate of 99.13%. The study by Oztekin et al. [21] used heat maps to explain deep learning-based models to physicians. The maps were created using the Grad-CAM method and applied to dental images. The dataset used was composed of 562 subjects labeled as caries and non-carious. The study employed data augmentation methods and chose Adam optimization, cross-entropy loss, 16 batch size, and a learning rate of 0.001. Two CNN-based models were used, with the ResNet model performing the best, achieving an accuracy of 92.00%, a sensitivity of 87.33%, and an F1-score of 91.61%.

The research undertaken encompasses all the necessary steps from data preparation and sorting to pre-processing. The data has been meticulously sourced and no ready-made data has been employed. The study employs four networks, with comparisons drawn among them. Additionally, two of the networks were trained from scratch while the other two were trained through the use of transfer learning, a novel combination for network training. The outcomes of the present study can serve as a valuable tool for dental practitioners to expediently and remotely diagnose caries. Additionally, this study can establish a correlation between artificial intelligence and dental science, leading to a more effective utilization of artificial intelligence in the field of dentistry. By utilizing the findings of this study and its subsequent enhancements, a diagnostic support system can be developed to identify dental caries.

The rest of the paper is organized as follows: In Section 2, materials and methods will be described. Evaluation metrics are defined in section 3.The results of the methods are illustrated and compared in Section 4. Finally, the discussion will be elaborated in Section 5.

## Materials and methods

In this section, we first introduce the dataset that was used in our investigations. Then, we describe the method including data mining steps applied to the dataset.

### Data acquisition

The identification of proximal and interproximal dental caries poses a challenge for dentists. To facilitate the detection of these areas, bitewing images have been identified as a more relatively accurate and suitable diagnostic tool. The rationale for this choice is grounded in the clarity of these images in capturing proximal and interproximal caries lesions. Notably, experts have provided guidance that bitewing images are better equipped to detect such lesions. A total of 713 Bitewing images were procured from the Samin Oral and Maxillofacial Radiology Center in Tehran, Iran. These images were saved in the JPEG format. Prior to analysis, a preprocessing step was deemed essential. The images were captured with the use of the Planmeca Periapical device, which is manufactured in Helsinki, Finland. During the imaging process, a Bitewing holder was employed to ensure accuracy and consistency. The imaging was performed using an 8 Ma, 60Kvp setting, and underexposure. Notably, patient information was not utilized during the process and the images were blindly analyzed (Despite the presence of patient information on each image, we refrained from utilizing any such data). Each image was captured in 0.128 seconds.

### Image pre-processing

**Fig. 1:**
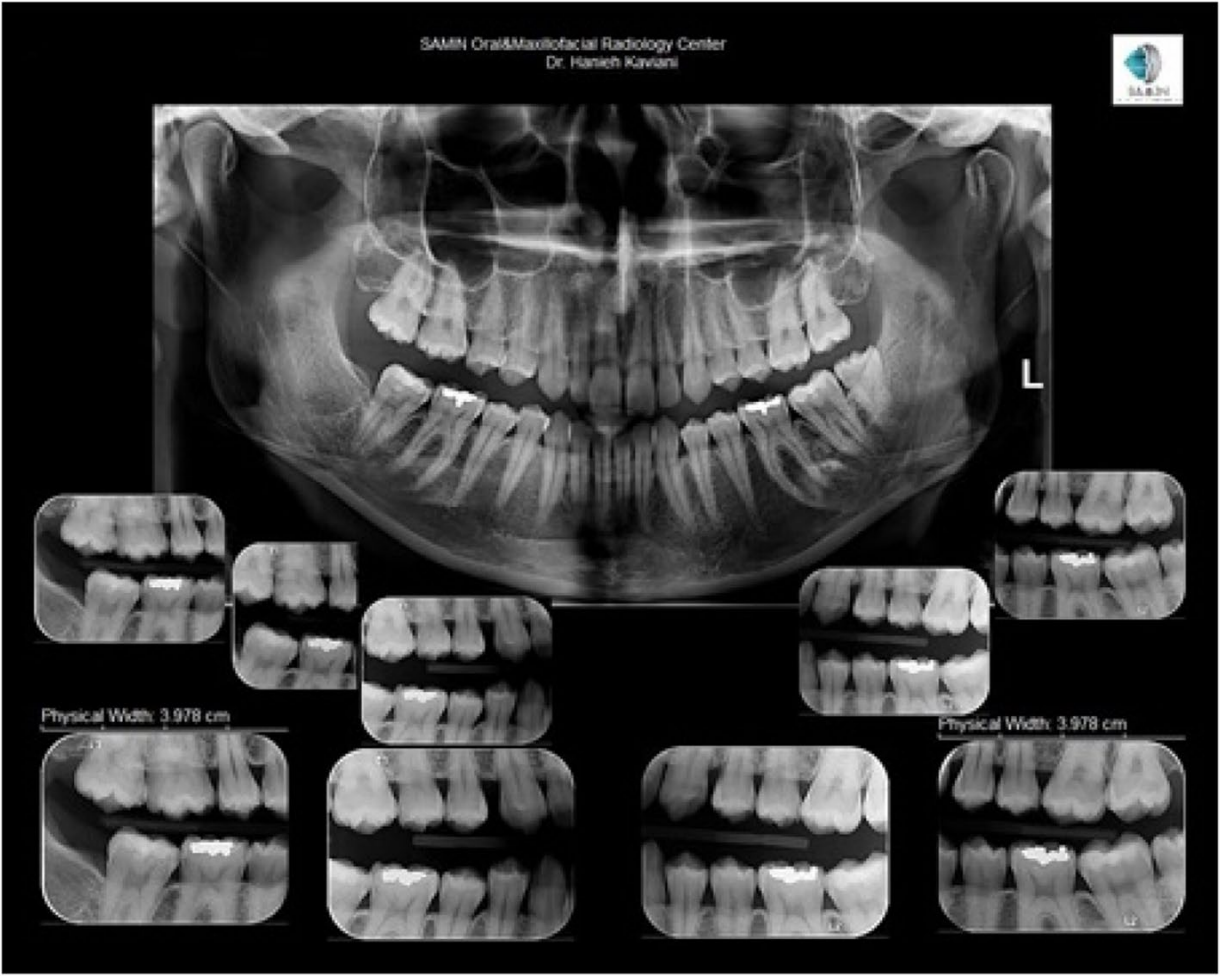
Raw image obtained from Oral and Maxillofacial Radiology Center

**Fig. 2:**
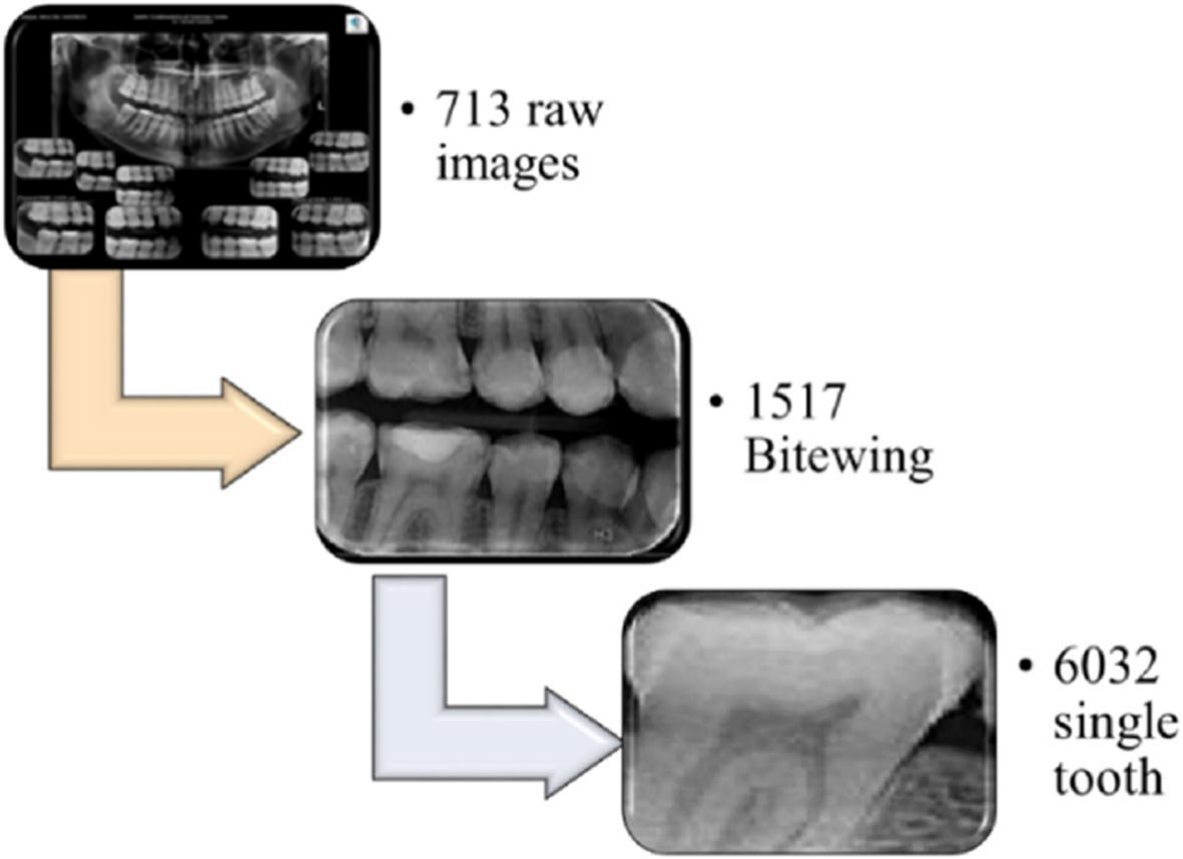
Pre-processing steps

A total of 713 Bitewing images were divided into smaller rectangular images, which are positioned beneath the panorama image in Fig. 1. These Bitewing images were then extracted using the Snipping tool, resulting in a total of 1517 images. To acquire single tooth images, all 1517 Bitewing images were cut using the Snipping tool, producing 6032 single tooth images. Next, all upper jaw images were rotated clockwise and segregated from the lower teeth. Finally, all single tooth images were extracted and resized to 100*100. For a visual representation of this process, refer to Fig. 2.

### Models’ architecture and model training

Machine Learning techniques include the extraction of features and the selection of proper features for a specific problem requires a domain expert [22]. Instead, Deep Learning which is a subset of machine learning that deals with the development of deep neural networks inspired by biological neural networks in the human brain [22], can automatically extract essential features from raw input data [23]. A convolutional Neural Network (CNN) is a type of deep and feedforward neural network. CNNs are designed to process data that come in the form of Grids or multiple arrays such as images, and their architecture is composed of several stages [24]. These networks have very efficient and popular applications in classification, image processing, and neural computer vision [25]. These networks are composed of three major layers. *Convolutional* layer, *Pooling* layer, and F*ully connected* layer. These layers can combine in different ways for feature extractions and this leads to the variation of CNN architectures. We used VGG16, VGG19, AlexNet, and ResNet50 in our study.

#### VGG16

**Fig. 3:**
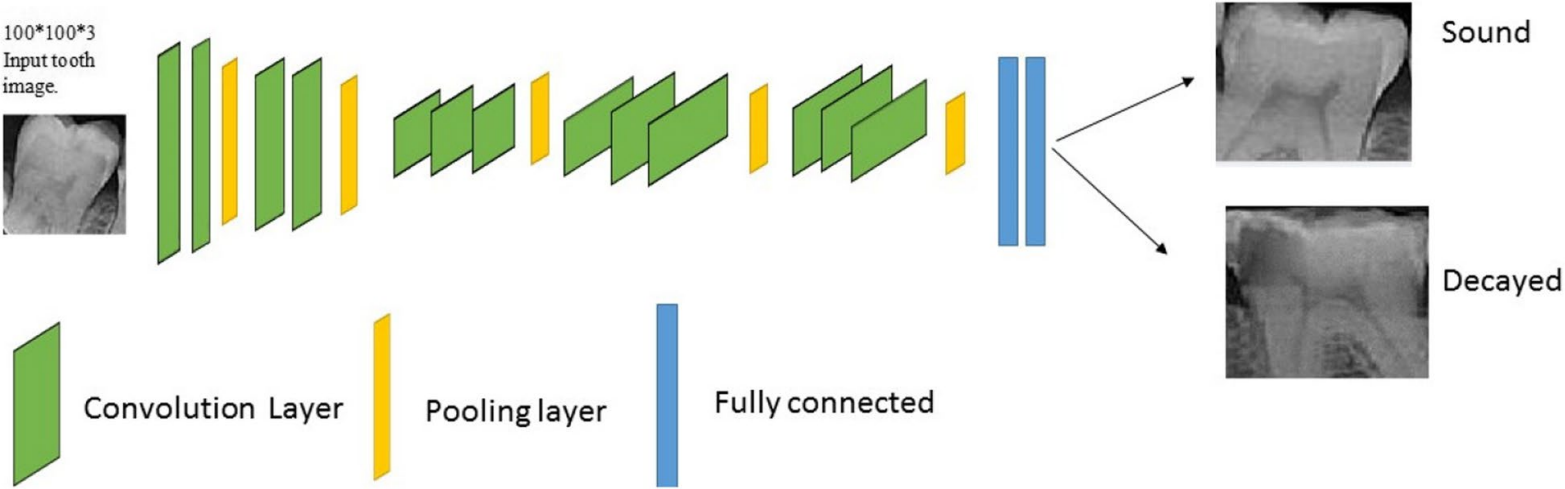
VGG16 architecture

This network was introduced by Simonyan and Zisserman in the Visual Geometry Group at Oxford University [26]. The input of this network is 224*224*3, however, we changed the model to feed 100*100*3 images in our research. The VGG-16 architecture consists of 16 layers, including 13 convolutional layers and 3 fully connected layers. Each convolutional layer uses a small receptive field of 3*3, and the stride is set to 1 pixel, with padding added to ensure that the spatial resolution is preserved. The max-pooling layer follows every two or three convolutional layers, which reduces the spatial resolution by half. The final three fully connected layers use the traditional neural network structure, with the first two layers having 4096 nodes, and the last layer having 1000 nodes corresponding to the number of classes in the ImageNet dataset. In our study, our problem is a two-class classification so 1000 changes to 2. The architecture is shown in Fig. 3. Number 16 means that it has a total of 16 layers that have some weights [27].

The list of parameters that are used in VGG16 is shown in Table 1.

**Table 1:** Layers and parameters of the VGG16 network

Layer	Output shape	Parameters
conv2d_1 (Conv2D)	(None, 100, 100, 64)	1792
conv2d_2 (Conv2D)	(None, 100, 100, 64)	36,928
max_pooling2d	(None, 50, 50, 64)	0
conv2d_3 (Conv2D)	(None, 50, 50, 128)	73,856
conv2d_4 (Conv2D)	(None, 50, 50, 128)	147,584
max_pooling2d_1	(None, 25, 25, 128)	0
conv2d_5 (Conv2D)	(None, 25, 25, 256)	295,168
conv2d_6(Conv2D)	(None, 25, 25, 256)	590,080
conv2d_7 (Conv2D)	(None, 25, 25, 256)	590,080
max_pooling2d_2	(None, 12, 12, 256)	0
conv2d_8 (Conv2D)	(None, 12, 12, 512)	1,180,160
conv2d_9(Conv2D)	(None, 12, 12, 512)	2,359,808
conv2d_10 (Conv2D)	(None, 12, 12, 512)	2,359,808
max_pooling2d_3	(None, 6, 6, 512)	0
conv2d_11(Conv2D)	(None, 6, 6, 512)	2,359,808
conv2d_12 (Conv2D)	(None, 6, 6, 512)	2,359,808
conv2d_13 (Conv2D)	(None, 6, 6, 512)	2,359,808
max_pooling2d_4	(None, 3, 3, 512)	0
flatten	(None, 4608)	0
dense	(None, 4096)	18,878,464
dense_1	(None, 4096)	16,781,312
dense_2	(None, 2)	8194
Total parameters: **50,382,658.** Trainable parameters: 50,382,658. Non-trainable parameters: 0.		

As shown in Table 1, VGG16 had 50,382,658 parameters to train.

#### VGG19

**Fig. 4:**
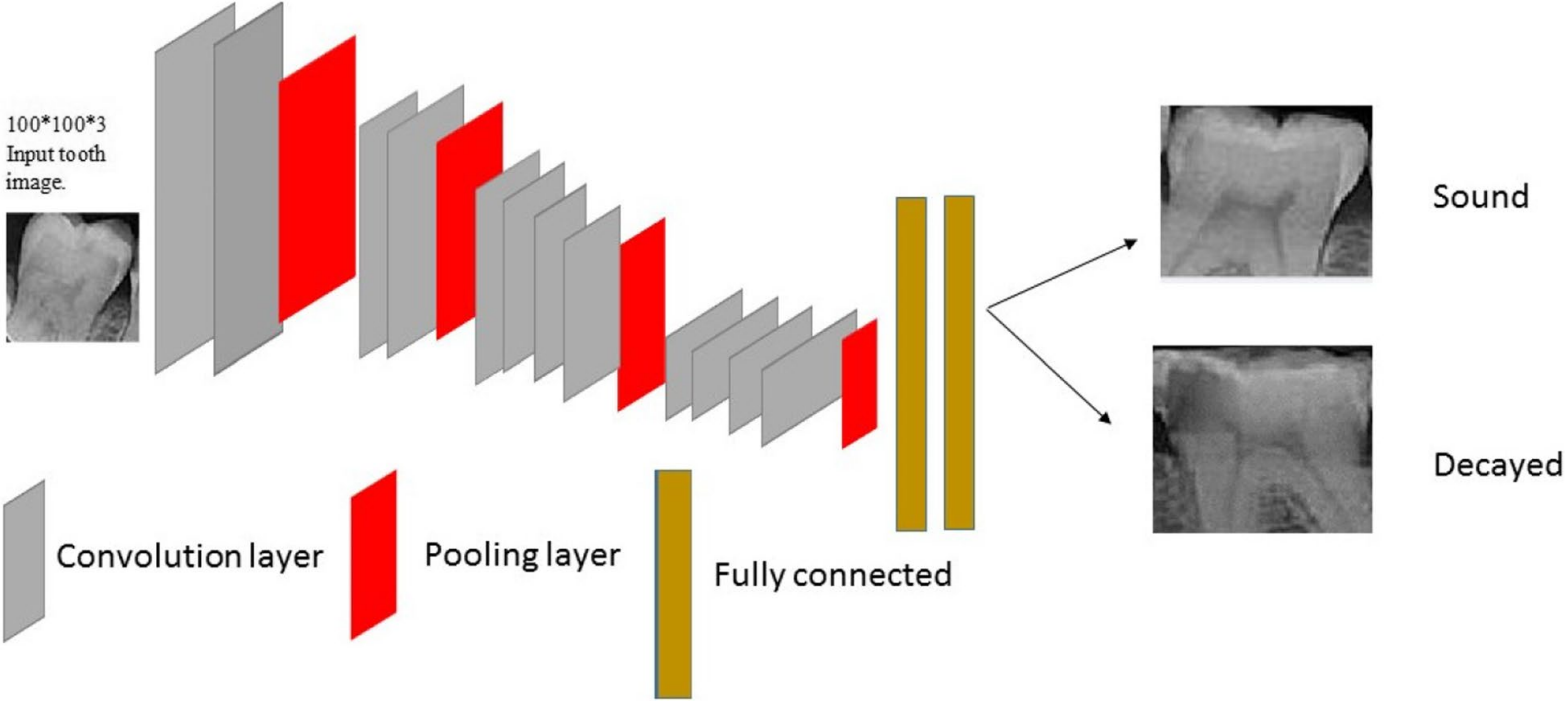
VGG18 architecture

Compared with traditional convolutional neural networks, it has been improved in network depth. It uses an alternating structure of multiple convolutional layers and non-linear activation layers, which is better than a single convolution The layer structure can better extract image features, use Maxpooling for downsampling, and modify the linear unit (ReLU) as the activation function, that is, select the largest value in the image area as the pooled value of the area [28]. The architecture of the network bears a resemblance to VGG16 but boasts three supplementary layers. A visual representation of this network can be found in Fig. 4.

#### AlexNet

**Fig. 5:**
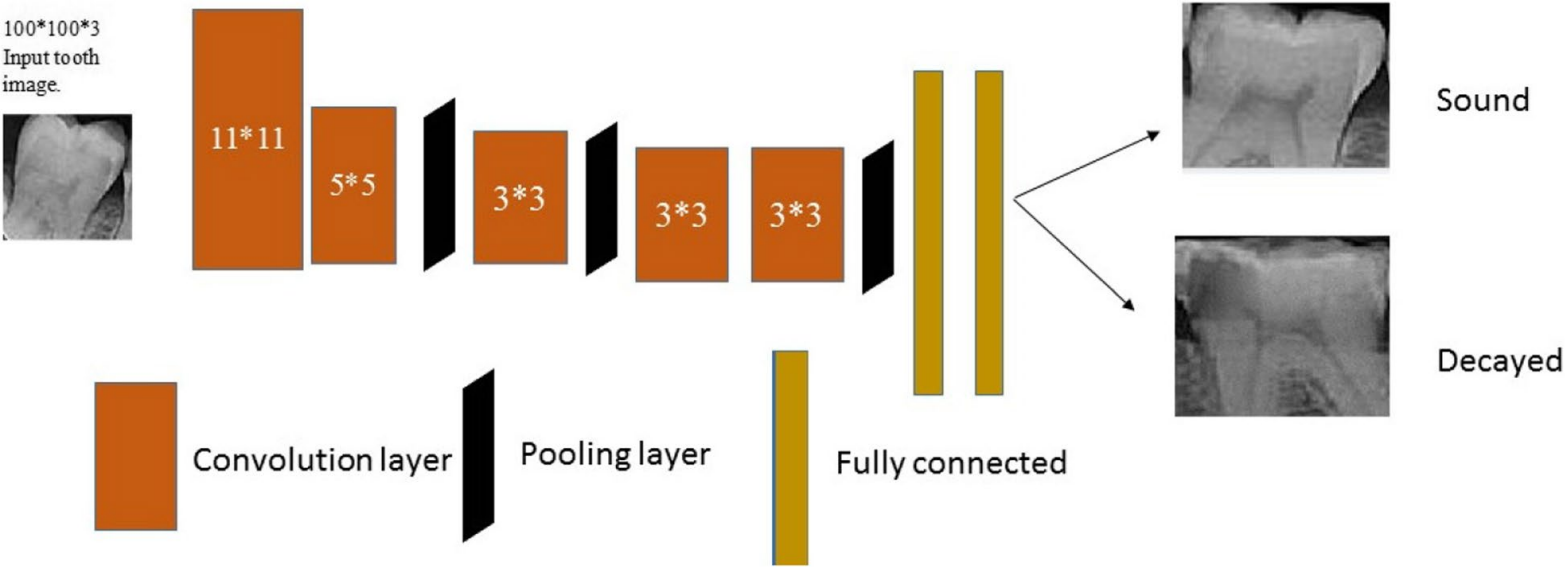
AlexNet architecture

This network was proposed by Krizhevsky et. Al. [29] in 2012. It achieved popularity in ILSVRC which was a challenge associated with the ImageNet classification. AlexNet was crucial for refocusing attention on deep learning research. Its architecture is similar to LeNet-5 but with additional layers and filters, resulting in a better extent and more learning variables [30]. AlexNet is a convolutional neural network that is 8 layers deep. The pre-trained network can classify images into 1000 object categories, such as keyboard, mouse, pencil, and many animals. As a result, the network has learned rich feature representations for a wide range of images. The network has an image input size of 227*227. In our case, the input size is 100-by-100 and the output is a binary classification, so 1000 changes to 2. The original paper’s primary result was that the depth of the model was essential for its high performance, which was computationally expensive but made feasible due to the utilization of graphics processing units (GPUs) during training [29]. The architecture of this network is shown in Fig. 5.

The parameters of this network are shown in Table 2.

**Table 2:** Layers and parameters of AlexNet network

Layer	Output shape	Parameters
conv2d_1 (Conv2D)	(None, 23, 23, 96)	34,944
batch_normalization_1	(None, 23, 23, 96)	384
max_pooling2d_1	(None, 11, 11, 96)	0
conv2d_2 (Conv2D)	(None, 11, 11, 256)	614,656
batch_normalization_2	(None, 11, 11, 256)	1024
max_pooling2d_2	(None, 5, 5, 256)	0
conv2d_3	(None, 5, 5, 384)	885,120
batch_normalization_3	(None, 5, 5, 384)	1536
conv2d_4 (Conv2D)	(None, 5, 5, 384)	1,327,488
batch_normalization_4	(None, 5, 5, 384)	1536
conv2d_5 (Conv2D)	(None, 5, 5, 256)	884,992
batch_normalization_5	(None, 5, 5, 256)	1024
max_pooling2d_3	(None, 2, 2, 256)	0
flatten_1 (Flatten)	(None, 1024)	0
dense_1 (Dense)	(None, 4096)	4,198,400
dropout_1 (Dropout)	(None, 4096)	0
dense_2 (Dense)	(None, 4096)	16,781,312
dropout_2 (Dropout)	(None, 4096)	0
dense_3 (Dense)	(None, 5000)	20,485,000
dropout_3 (Dropout)	(None, 5000)	0
dense_4 (Dense)	(None, 2)	10,002
Total parameters: **45,227,418.** Trainable parameters: **45,224,666.** Non-trainable parameters: **2752.**		

As shown in Table 2, AlexNet had 45,224,666 parameters to train, 2752 hyper-parameters, and 45,227,418 total parameters.

#### ResNet

The deepness of a network is a crucial point. Evidence has shown that deeper networks are beneficial and can lead to better results. This is useful to prevent gradient problems before they occur. As networks get deeper, the risk of gradient issues increases. This vanishing/exploding gradient might hinder the convergence of the network to the actual results [3]. It has several types varied by the number of layers, in the current study, ResNet-50 was used. We used pre-trained ResNet50 for this study. ResNet-50 is a deep convolutional neural network (CNN) architecture that was introduced by Kaiming He et al. in 2015. It is part of the ResNet (Residual Network) family of models, which were developed to address the problem of vanishing gradients and degradation of performance as the network depth increases. The ResNet-50 architecture consists of 50 layers, including convolutional layers, batch normalization layers, activation functions, and fully connected layers. The key innovation of ResNet-50 is the introduction of residual connections, which allow the network to learn residual mappings instead of directly learning the desired underlying mappings. These residual connections address the degradation problem by facilitating the flow of gradients through the network, enabling deeper models to be trained more effectively.

These networks utilize the skip connection and residual block for solving the gradient problems.

### Exprimental setup

In Table 3, all the hyperparameters which are used are listed. Table 4 lists all the information on the software/hardware used in this study.

**Table 3:** Value of the hyperparameters

Hyperparameters	Value
**Loss function**	Sparse categorical entropy
**Optimizer**	Adam
**Learning rate**	9*10^−5^
**Input size**	100*100*3
**Epochs**	300

**Table 4:** Software or hardware information

Soft/Hardware	Information
**Programming language**	Python
**Platform**	Google Colab pro
**GPU**	Tesla V100 – SXM2 16GB
**RAM (Storage)**	25 GB (100 GB)
**Most used packages**	Numpy, Keras, Tensorflow, and glob

Experimentally, the best learning rate for this study is defined as 9*10^−5^ and 300 epochs for training.

The study employed Python as the programming language. To obtain the necessary features for deep learning and convolutional neural networks, the Keras and TensorFlow libraries were utilized. Since the input data came in matrix form, specific libraries such as Numpy and Glob were employed for data manipulation. Implementing Python programming language can be done on various platforms. However, deep learning algorithms require high storage capacity and strong hardware. In this study, a reliable platform for Python programming in deep learning was Google Colab, which offers a robust cloud GPU and storage. Hardware and software details can be found in Table 4.

Our endeavor was to design a Clinical Decision Support System (CDSS) software that could effectively and precisely diagnose dental caries. In order to achieve this goal, we conducted a comprehensive assessment and comparison of different Convolutional Neural Network (CNN) model architectures.

**Fig. 6:**
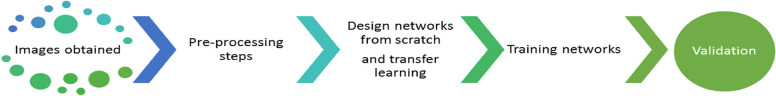
Diagram of the steps

In Fig. 6, The process flow from the initial phase to the final phase has been concisely outlined.

## Evaluation metrics

Our study focused on classifying Bitewing images as either sound or decayed teeth, using four prominent CNNs: VGG16, VGG19, AlexNet, and ResNet-50.In this section, we shall present a comprehensive account of the experimental results and the comparative analysis of the evaluation metrics for each of the CNN model architectures. The metrics under consideration are Confusion matrix, Accuracy, Precision, Sensitivity (Recall), Specificity, and F1-Measure. We shall begin by defining each metric to provide a clear understanding and context for our findings. Our primary objective is to identify the most effective CNN model architecture for the accurate diagnosis of dental caries in Bitewing images. *Confusion matrix.*

Confusion matrices represent counts from predicted and actual values. The output “TN” stands for True Negative which shows the number of negative examples classified correctly (Actual sound tooth which correctly classified sound). Similarly, “TP” stands for Predicted Actual for True Positive which indicates the number of positive examples classified accurately (Actual decayed tooth which is classified as decayed correctly). The term “FP” shows a False Positive value, i.e., the number of actual negative examples classified as positive (Actual sound tooth which wrongly classified as decayed); and “FN” means a False Negative value which is the number of actual positive examples classified as negative (Actual decayed tooth which is wrongly classified as sound) Using the aforementioned terms, several evaluation criteria can be made.

### Accuracy

Accuracy is a metric obtained by dividing the number of correctly classified by the total number of cases. This can be measured as the below formula.

1 $$Accuracy=\frac{TP+ TN}{TP+ TN+ FP+ FN}$$

### Precision

In medical applications of machine learning, a lack of data leads to an imbalanced dataset that can question the accuracy metric. In this case, other metrics can be used because they can be calculated for each class and imbalances would not affect them. In the context of evaluating machine (deep) learning algorithms., precision for a class refers to the number of correctly classified items (correctly labeled) divided by the sum of either true or false items labelled as belonging to that class. This can be calculated as below.

2 $$Precision=\frac{TP}{TP+ FP}$$

### Sensitivity

Sensitivity (Recall) is an important metric used in a machine (deep) learning model to assess if the model is performing successfully. Sensitivity is the probability that a positive result occurs given that the sample is indeed positive [31]. That is to say that, Sensitivity refers to a model’s ability to designate an individual with a disease (tooth decay) as positive. A highly sensitive test means that there are few false negative results, and thus fewer cases of disease (tooth decay) are missed. In our case, it can be defined as the probability that a decayed tooth is truly classified. It can be calculated as below.

3 $$Sensitivity\ \left( Recall\ or\ TPR\right)=\frac{TP}{TP+ FN}$$

### Specificity

Another important metric is specificity. This criterion refers to the probability of a negative result given that the sample is negative. That is to say that, the probability of truly classified a sound tooth. In other words, the specificity of a model is its ability to designate an individual who does not have a disease (tooth decay) as negative. A highly specific test means that there are few false positive results. It may not be feasible to use a model with low specificity for screening since many teeth without the caries will screen positive, and potentially receive unnecessary diagnostic.

4 $$Specificity\ \left(\ FPR\right)=\frac{TN}{TN+ FP}$$

### F (1)-score

The compound of precision and sensitivity (Recall) is defined as F1-measure or F-measure. The F-1 score is defined as the harmonic mean of precision and sensitivity. The value of this measure for a classification algorithm is equal to 1 under the ideal condition and equals zero under the worst condition.

5 $$\textrm{F}(1)\_ score=2\ast \frac{Precision\ast Sensitivity}{Precision+ Sensitivity}$$

## Results

**Fig. 7:**
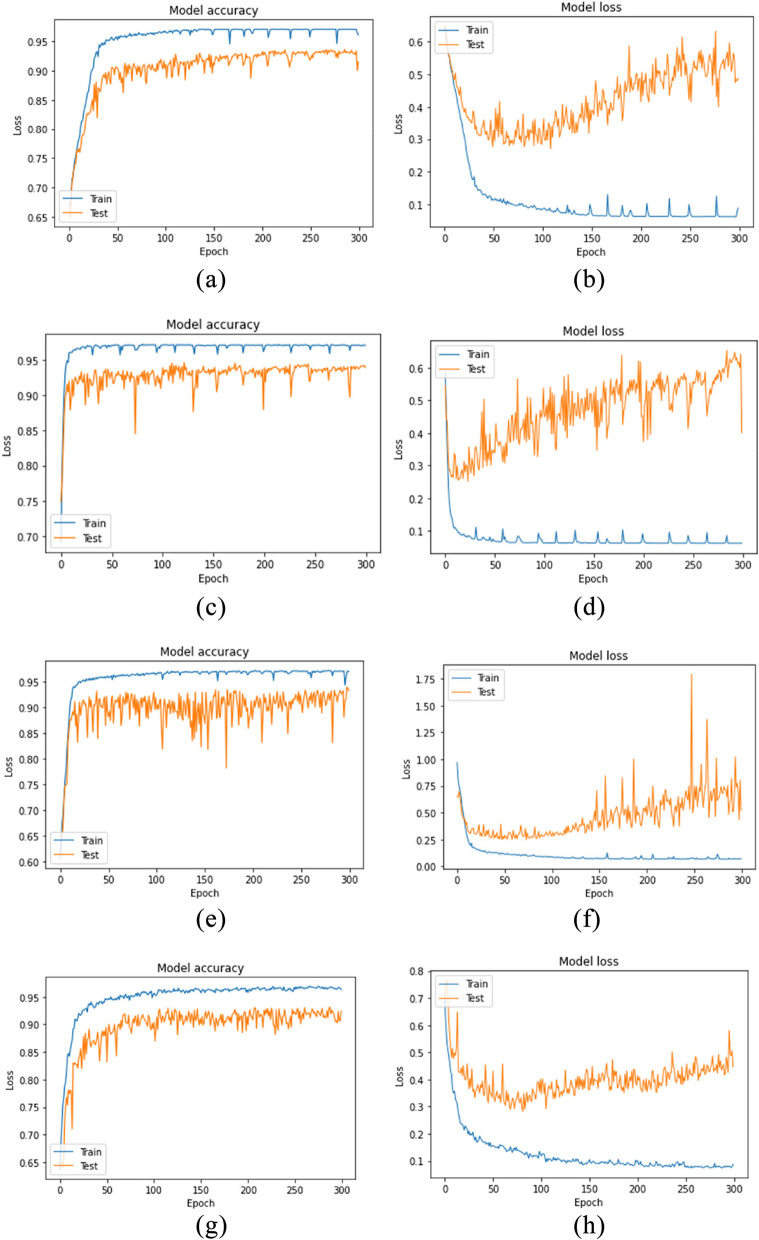
Model training and validation results for four different models over 300 epochs: VGG16: (**a**) is model accuracy and (**b**) is loss over each epoch. VGG19: (**c**) is Model accuracy and (**d**) is loss over each epoch. AlexNet: (**e**) is model accuracy and (**f**) is loss over each epoch ResNet50: (**g**) is model accuracy and (**h**) is loss over each epoch

In Fig. 7 the training of the model accuracy and loss of VGG16, VGG19, AlexNet, and ResNet-50 methods is depicted. All accuracy graphs had an ascending trend for both training and test data. Although the loss value had some oscillations in epochs, it recorded a descending trend. The graphs are presented separately for the Train and Test data sets. In graph a, which represents the VGG 16 model, the accuracy has demonstrated a significant increase from 65% to over 95% in the last epoch. The accuracy of the Test data set has also improved from 65% to over 90%. Graph b illustrates the output of the categorical cross-entropy function for loss. For the train data set, the loss has decreased from more than 0.6 to approximately 0, and the Test data set’s loss has also decreased from more than 0.6 to around 0.4. Both data sets show a consistent downward trend for each epoch, although the reduction for the Test data set is marginally less than the Train data set. Nonetheless, it reflects a similar downward trend. Diagrams c and d depict the accuracy and loss per epoch for the VGG 19 model, which are similar to the previous diagram. Notably, the accuracy of the training data set has exhibited significant improvement, rising from below 70% to nearly 100%, with minor fluctuations in accuracy levels. Similarly, the graph for the test data set has also shown improvement, increasing from under 70% to roughly 94%, albeit with more fluctuations and a lower amount in comparison to the training data set. In graph d, the loss has declined from around 1 to zero for the training data set. However, for the test data set, the loss has decreased from 1 to approximately 0.4, despite fluctuations. The AlexNet model has exhibited significant improvements in accuracy, particularly in the training dataset where it has increased from 60% to over 95% with minimal fluctuations. The test dataset has also shown improvement, increasing from 60% to over 90%. Notably, the loss in the training dataset is almost negligible, while the test dataset experiences significant fluctuations around epochs 170, 250, and 260. Although the loss can sometimes reach 1.75, it gradually decreases over time and reaches 0.5 in the final epoch. The ResNet-50 model is illustrated through two graphs, labeled as g and h. The first graph, g, presents a notable improvement in the accuracy level of the train data set, escalating from below 65% to over 95%. Furthermore, the accuracy of the test data set has also witnessed an improvement from below 65% to approximately 90%. In contrast, the h chart demonstrates the loss rate for both the train and test data sets. The loss rate for the train data set has decreased from 0.8 to 0, whereas for the test data set, it has increased from 0.8 to approximately 0.5.

**Fig. 8:**
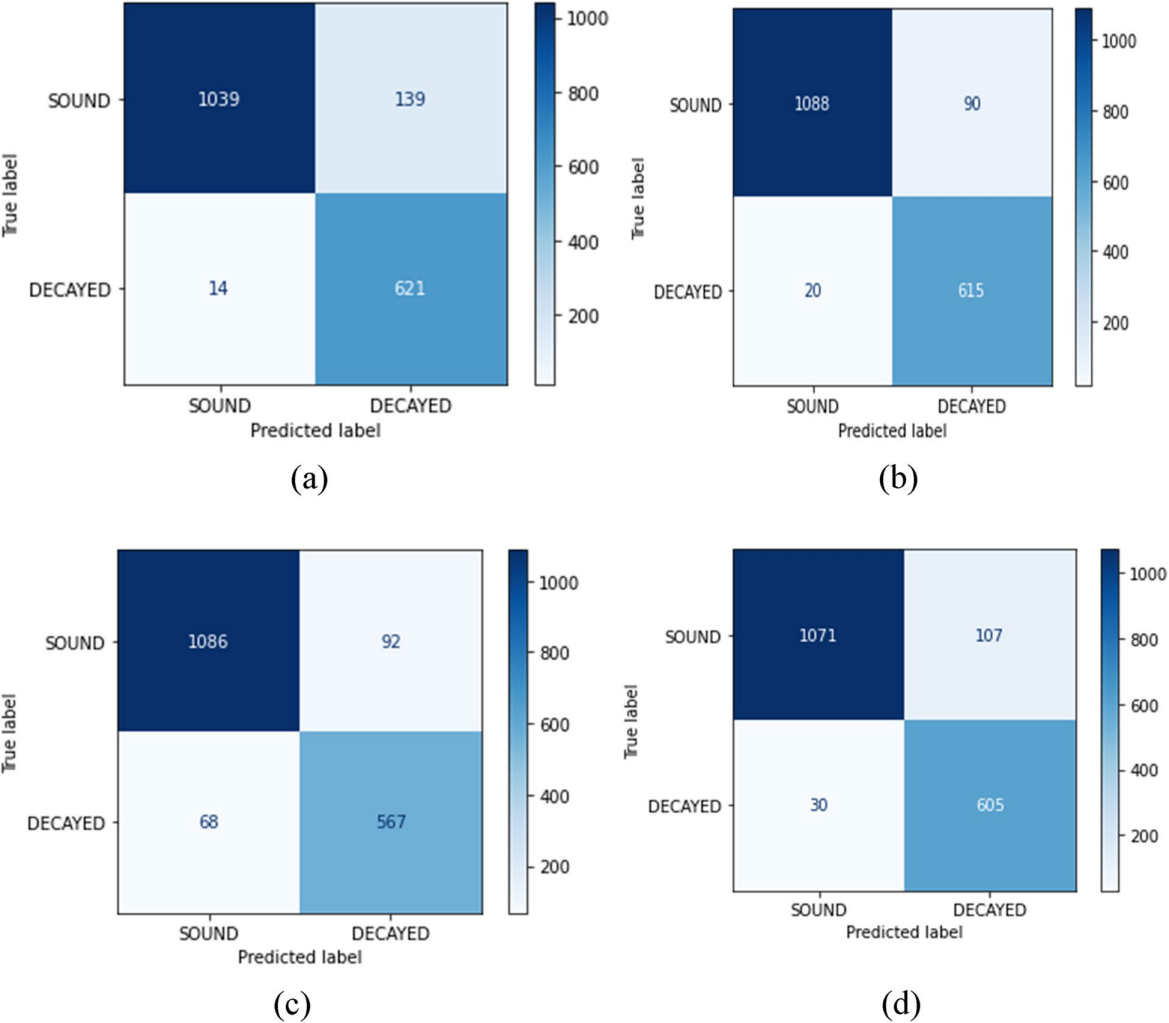
The measure of TP, TN, FN, and FP of the **a**: VGG16, **b**: VGG19, **c**: AlexNet, and **d**: ResNet50, which forms confusion matrices

In medical applications, it is of great importance to minimize the false negative and false positive outcomes. These values are considered errors and can affect the evaluation metrics. They can also have effects on the decisions of doctors and medical practitioners. The confusion matrix of the models is represented in Fig. 7. When analyzing data using a confusion matrix, it’s important to understand how the predicted and actual values are represented. The X-axis displays the predicted values, while the Y-axis represents the actual values. The upper left quadrant of the matrix represents the true negative (TN) values, which occur when the prediction is negative and the actual value is also negative. The upper right quadrant represents false positive (FP) values, which occur when the prediction is positive but the actual value is negative. The bottom left quadrant represents false negative (FN) values, which occur when the prediction is negative but the actual value is positive. Finally, the bottom right quadrant represents true positive (TP) values, which occur when the prediction is positive and the actual value is also positive. Understanding these values is crucial when interpreting the results of a confusion matrix. Figure 8 displays the confusion matrices for all models evaluated, including VGG16, VGG19, AlexNet, and ResNet-50, identified in Fig. 8 as a, b, c, and d, respectively. Notably, VGG19 demonstrated the highest TN value, reaching 1088, while VGG16 had the highest FP value, totaling 139. AlexNet, on the other hand, exhibited the highest FN value, at 68, while VGG16 achieved the highest TP value, reaching 621. These numerical values offer valuable insight into the efficacy of each model in properly classifying TP, FP, TN, and FN.

All networks well performed in terms of reducing the number of false negatives and false positives, but VGG19 was superior to other networks in this study.

In Table 5, the overall accuracy, precision, sensitivity, specificity, and F1-Score are shown.

**Table 5:** The overall evaluation metrics for VGG16.VGG19, AlexNet,andd ResNet50

Network	Accuracy	Precision	Sensitivity	Specificity	F1-score
VGG16	92%	90%	93%	**97%**	91%
VGG19	**94%**	**93%**	**95%**	96%	**93%**
AlexNet	91%	90%	91%	89%	90%
ResNet50	92%	91%	93%	95%	92%

According to the findings in Table 5, VGG19 demonstrated the most accurate performance out of all the networks utilized in the study. All of the models exhibited high levels of accuracy, exceeding 90%, which suggests that they effectively accomplished their respective tasks. VGG19 stood out with the highest precision at 93%, rendering it the most sensitive model for identifying decayed teeth. On the other hand, VGG16 demonstrated the highest specificity at 97%, making it more adept at identifying sound teeth. VGG19 also scored the highest F1-Score at 93%. Overall, VGG19 achieved the top marks in four crucial criteria, making it the most outstanding model in the study. For a more detailed analysis, please refer to Table 6, which provides an overview of the evaluation metrics for each class.

**Table 6:** Precision, Sensitivity, and F1-Score of two classes of each network

Class	Sound			Decayed		
Network	Precision	Sensitivity	F1-Score	Precision	Sensitivity	F1-Score
**VGG16**	0.99	0.88	0.93	0.82	0.98	0.89
**VGG19**	0.98	0.92	0.95	0.87	0.97	0.92
**AlexNet**	0.94	0.92	0.93	0.86	0.89	0.88
**ResNet50**	0.97	0.91	0.94	0.85	0.95	0.90

**Fig. 9:**
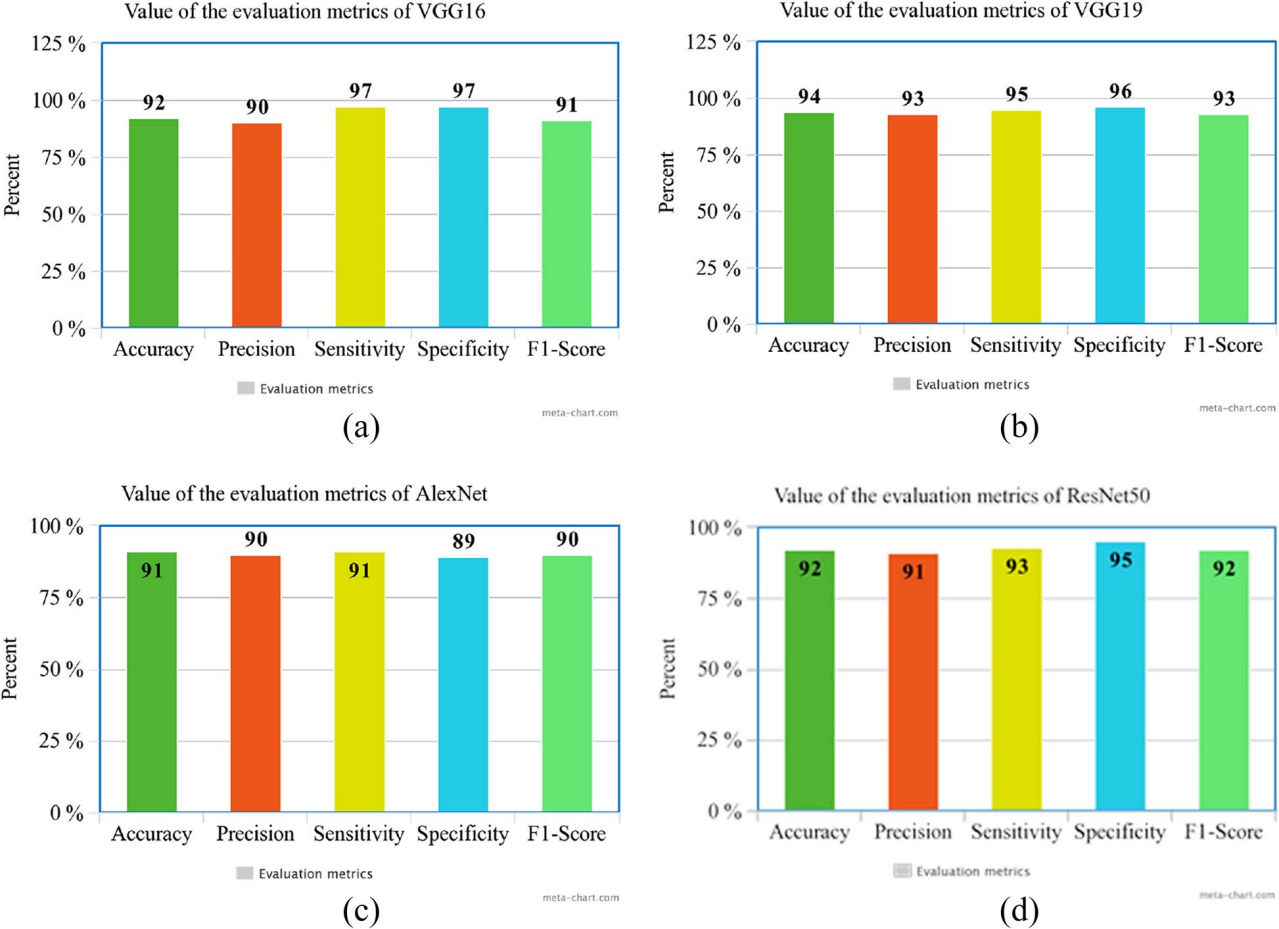
Evaluation metrics values for the following models: **a** VGG16, **b** VGG19, **c** AlexNet, and **d** ResNet50

As shown in Fig. 9, all of the values of the evaluation metrics for all networks are shown.

To draw a comparison between the current study and similar ones, we have limited our selection to studies that employed Bitewing images and one or more VGG16, VGG19, AlexNet, and ResNet50 architectures. Due to the exclusive processing of our data within Iran, we were unable to compare our findings with those of other studies based on data, as we lacked access to other datasets. In this section, we aim to only highlight the differences in evaluation metrics and sample size between our study and other similar studies [3, 18]. are selected for comparison because they meet the criteria of using the same models as our study and also Bitewing. Table 7 demonstrates the differences between the current study and [3].

**Table 7:** Comparison of the accuracy of the current study and [3]

Model/sample size	[3]	Current study
**VGG19**	80.25%	93.93%
**AlexNet**	90.30%	91.17%
**ResNet-50**	82.72%	92.44%
**Sample size**	278	1517

In [3], VGG19, AlexNet, and ResNet50 were common in the current study. They used another network too and the details are shown in the literature review section.

The findings presented in Table 7 demonstrate that the current study achieved higher accuracy in all networks when compared to the previous study [3]. However, it would not be appropriate to conclude that our work is superior to theirs as we did not utilize an identical dataset. In the event that we used the same dataset, we would be able to make a more definitive comparison. Table 8 presents a comparison between our study and [18], who also employed the VGG16 network. The precision, sensitivity, and F1-score of both investigations are outlined in Table 8.

**Table 8:** Comparison of the current study and [18]

Study	Precision	Sensitivity (recall)	F1-Score
Current study	82%	98%	89%
[18]	84%	84%	84%

The architecture of pre-trained neural networks such as ResNet-50,VGG19, VGG16, and AlexNet are defined, so different results can be obtained by changing the parameters and hyper-parameters of these networks. We achieved higher evaluation metrics than those of [3, 18]. some studies were extracted from [32] to compare our work with them.

Table 9 presents the accuracy of studies that reported this criterion in the introduction section, irrespective of the image type used. These results were compared to that of the current study. It is notable that studies not mentioned in this section but listed in the similar works section did not report the accuracy criterion value. The most comparable study to the current one is [3]. Moreover, the current study showed acceptable accuracy when compared to other similar works.

**Table 9:** Comparison of current study networks with related works

	[10]	[11]	[12]	[13]	[14]	[3]	[15]	[16]	[17]	[19]	[20]	[21]	Current study
VGG16													**92**
VGG19						80.25%							**94%**
ResNet50						82.72%						92%	**91%**
AlexNet					96%	90.30%							**92%**
Inception			82%				73.3%						
GoogleNet						87.04%							
DenseNet	88.4%												
ANN		98.8%											
LA-PSO				99%									
FCNN								94%					
YOLOv3									94.59%				
DNN										86.1%			
DCNNE											99.13%		

To draw a comparison between the efficacy of traditional methods and Convolutional Neural Networks (CNNs), a specific traditional method shall be elucidated. In [33], the specificity of the Bitewing for the detection of dental caries was 87.7%. Compared with CNNs in this study, all of the networks achieved higher specificity. It’s worth noticing that the sensitivity observed in the current study was significantly higher than the sensitivity reported in [33, 34] acquired 76% for digital and 75% for conventional radiography which is lower than the proposed method in this study [35]. showed 50 to 70% sensitivity in detecting caries from Bitewing with conventional methods. In [16] it is noted that AI outperforms conventional methods.

## Discussion and conclusion

The primary aim of the research conducted was to develop models that could automatically detect dental caries from Bitewing images. The pre-trained models included VGG19 and ResNet50, as well as VGG16 and AlexNet through transfer learning. The results revealed that VGG19 achieved the highest accuracy rate (94%) amongst other CNNs. This model outperformed other models used in the research and can be utilized as a preliminary model for detecting tooth decay using deep learning. The AI-based diagnostic system devised in this research has the potential to support dental professionals in diagnosing decayed teeth with high precision and promptly, thereby minimizing the need for frequent dental visits for check-ups. This research is a cost-effective and time-saving approach for both patients and dental professionals while ensuring acceptable accuracy in diagnosing tooth decay. Medical research frequently faces challenges due to limited data, resulting in a reduction in the accuracy and quality of research. The shortage of data is a significant hurdle in interdisciplinary studies, particularly when medical science is combined with deep learning algorithms that require a significant quantity of data to converge to an appropriate accuracy for detection or classification. The inadequacy of data in medical problems can have a detrimental effect on the quality of research in this field and necessitates attention for future research.

In this research, an acceptable amount of data was procured from the Samin Maxillofacial Radiology Center, Tehran, Iran. The research encountered several obstacles, including the number of images and the requirement for a system with robust hardware to train deep learning algorithms. Every deep learning algorithm necessitates a significant amount of time and storage for training, which necessitates more powerful hardware resources. This can be a costly undertaking and requires technical expertise. In this study, four deep neural networks were trained. Without the aid of resources such as Google Colaboratory, training these networks would have been arduous and time-consuming, and the networks may not have been fully trained. Studies based on deep learning face the challenge that the mentioned tool can solve this challenge to a good extent. If the amount of data and network parameters are so large that even a tool like Google Colab does not respond, it is necessary to use extremely powerful hardware. Another challenge of this study was defining classes to distinguish healthy teeth from decayed teeth in the training dataset, which was a time-consuming process due to the large volume of data. Of course, this challenge was solved to a great extent with the help of a specialist dentist and a radiologist assistant, and it took less time. This challenge can be solved in future research by referring to dental science. Another challenge was the cropping stage of single tooth images. Due to the very close proximity of the teeth, it was difficult to crop the images accurately and correctly. This challenge was also solved by using maximum precision for cropping images. Due to the variety of dental images and their different usage, it is vital to choose the images correctly. In this study, according to the opinion of consultants, dentists, and maxillofacial radiologists, it was recommended to use the Bitewing image to detect tooth decay, especially approximal and interproximal caries lesions.

Other images are also used to detect caries, which should be selected according to the opinion of experts on the type of image. This research, in addition to helping dentists, will cause more cooperation between dentists, computer science experts, and data science experts. The interdisciplinary nature of this research can generate new research in the field of dentistry. Among these combinations, it can include the diagnosis of other oral and dental diseases or even the jaw, like oral cancers. This research can even directly and indirectly help patients. For example, patients may prefer not to see a dentist for a simple tooth decay diagnosis. This decision may be due to both financial and time aspects. Based on current research and comparable studies, it is possible for dentists and patients alike to ascertain the condition of a tooth as either healthy or decayed through the use of simplified and user-friendly CNNs, which can be utilized by even the most inexperienced of individuals. On the other hand, due to the busyness of patients, a simple examination may not be feasible for them, this may cause a delay in going to the dental office, which can cause progress or recurrence of tooth decay because of the nature of this disease. During the Covid-19 pandemic and the worldwide quarantine, the importance of having such a diagnostic system that did not require a visit to the dentist’s office was overt. By using this system, the diagnosis is done at an appropriate time and an acceptable velocity, and the patient only goes to the dentist for treatment. It is important to mention that this research or even similar research alone cannot take the place of the dentist’s opinion, but it can act as an intelligent assistant and help the dentist in diagnosis. Using the results of these studies does not mean abandoning the opinion of dentists. In this research, we were only looking for whether a tooth is carious or not. But another application of these networks or artificial intelligence, in general, can be mentioned as the use of networks to find the exact location of caries or even their severity, which can be investigated in future research.

In this research endeavor, we conducted a comprehensive comparative analysis of four distinct network models, utilizing a specific dataset. As per our established criteria, the VGG19 model showcased the highest level of accuracy amongst the four networks that were evaluated. It is noteworthy to mention that two of the aforementioned networks were pre-trained (VGG19 and ResNet50), while the other two were trained from scratch. While we attempted to compare our findings with similar studies, we encountered a significant variance in image types, which prevented us from making a direct comparison. One of the most intriguing findings of our study was that the model with the highest accuracy in another study produced the least accuracy in our analysis, and vice versa. This particular finding underscores the critical importance of recognizing that a model’s performance is entirely contingent on the conditions of the study and cannot be universally assumed to be the best. Several factors, such as differences in the dataset, network architecture, and hyper-parameters, can significantly influence a model’s effectiveness. Therefore, future research endeavors must take these factors into account carefully to ensure accurate and reliable results. In light of the research that has been conducted, it is advised that future investigations take into account the following recommendations.

The objective of this study is not to supplant human agents with artificial intelligence. Rather, this research aims to introduce an assistant for dentists that can diagnose caries with a reasonable degree of accuracy. The replacement of human agents with artificial intelligence is a delicate process that necessitates numerous studies. Furthermore, if artificial intelligence is to replace human agents, it would be restricted to the diagnostic stage and not the treatment stage. From a moral standpoint, patients have the right to receive an accurate diagnosis and appropriate treatment for their condition. This can be achieved when artificial intelligence is sufficiently advanced to comprehend these cases or when there is a second stage in which the AI’s findings are verified by a dentist or physician. One of the challenges of replacing human agents with artificial intelligence is that patients may not be able to perceive emotions and sentiments as well from artificial intelligence, which may disrupt the transmission of the patient’s and the physician’s characteristics. It is improbable that patients will embrace the concept of “machine-human” medical relationships in lieu of “human-human” interactions [36].

Artificial intelligence models require continuous monitoring and maintenance to ensure that they remain accurate and effective. This process entails performing ongoing data quality checks, updating models with new data, and retraining models as necessary. The significance of monitoring and maintaining AI models lies in the fact that they are highly dependent on the data they were trained on, and as new data becomes available, models must be adjusted accordingly to remain relevant and effective [37]. Regularly monitoring and maintaining AI models ensures that their accuracy and effectiveness remain consistent and that they continue to deliver reliable results. Failure to perform regular monitoring and maintenance could lead to reduced accuracy, reliability, and effectiveness, which could significantly impact business operations and decision-making processes. Therefore, it is imperative to prioritize the ongoing monitoring and maintenance of AI models to maximize their potential and deliver optimal outcomes.

The present study aims to analyze the condition of healthy and decayed teeth, with the potential to include a third category, i.e., restoration. The severity of decay can be classified into different levels, ranging from surface-level decay to the most severe form. While the study does not cover the use of neural networks for treating caries, it proposes the establishment of classes to choose the appropriate treatment and recommend the best course of action. However, it is imperative to seek detailed advice from experienced dentists to minimize any errors in the treatment process. The outcomes of the study could be applied through a mobile application that facilitates users, including those unfamiliar with caries, in uploading images of their teeth to determine their status of dental health.

## Data Availability

The datasets generated and/or analysed during the current study are not publicly available due to the importance of privacy but are available from the corresponding author on reasonable request.
